# Polypharmacy with potentially inappropriate medications as a risk factor of new onset sarcopenia among community-dwelling Japanese older adults: a 9-year Kashiwa cohort study

**DOI:** 10.1186/s12877-023-04012-y

**Published:** 2023-06-26

**Authors:** Tomoki Tanaka, Masahiro Akishita, Taro Kojima, Bo-Kyung Son, Katsuya Iijima

**Affiliations:** 1 Institute of Gerontology, The University of Tokyo, Tokyo, Japan; 2 Department of Geriatric Medicine, Graduate School of Medicine, The University of Tokyo, Tokyo, Japan; 3 Institute for Future Initiatives, The University of Tokyo, Tokyo, Japan

**Keywords:** Polypharmacy, Potentially inappropriate medication, Muscle mass, Physical function

## Abstract

**Background:**

Clinical evidence demonstrating a longitudinal association between prescribed medications and sarcopenia onset is lacking. We investigated the association of polypharmacy (the use of five or more medications) and potentially inappropriate medications (PIMs) with sarcopenia risk in community-dwelling older adults.

**Methods:**

In this longitudinal population-based cohort study, 2,044 older residents with no long-term care needs were randomly selected from a community in Kashiwa, Japan. Baseline data collection was conducted in 2012, with follow-ups in 2013, 2014, 2016, 2018, and 2021. Prescribed medications and PIMs (drugs listed in the Screening Tool for Older Person’s Appropriate Prescriptions for the Japanese or potentially muscle-wasting drugs) were identified through interviews. New-onset sarcopenia was identified according to the 2019 criteria of the Asian Working Group for Sarcopenia over a 9-year period and analyzed. We used Cox proportional hazards models to test the longitudinal association of prescribed medications with sarcopenia onset.

**Results:**

Of the 1,549 participants without sarcopenia at baseline (mean age, 72.5 ± 5.5 years; 49.1% women; median and interquartile range, 6.0 [4.0–9.0] years), 230 experienced new-onset sarcopenia during the follow-up. After adjusting for confounders, polypharmacy combined with PIM use was strongly associated with new-onset sarcopenia (adjusted hazard ratio, 2.35; 95% confidence interval, 1.58–3.51; *P <* 0.001). No significant associations were observed for either PIM use or polypharmacy alone.

**Conclusions:**

Polypharmacy combined with PIM use, but not polypharmacy alone, was associated with an increased risk of new-onset sarcopenia over the 9-year follow-up period among community-dwelling older adults. Limiting polypharmacy and imposing the prescription of appropriate medications may facilitate sarcopenia prevention.

**Supplementary Information:**

The online version contains supplementary material available at 10.1186/s12877-023-04012-y.

## Background

Sarcopenia, a common and serious disorder that shortens healthy life expectancy in aging populations, such as those in Japan [1], involves the progressive loss of skeletal muscle with increased risks of adverse health outcomes, including frailty, functional disability, and mortality [1–3]. While the prevalence of sarcopenia varies depending on the assessment method and criteria, it is about a little more than 10% among Japanese community-dwelling older adults [4]. Sarcopenia was assigned an individual International Statistical Classification of Diseases and Related Health Problems code (M62.84) in 2016, with Japan publishing clinical practice guidelines in 2018. Considering the aging global population, sarcopenia is increasingly becoming a health care priority.

Adverse drug events have become increasingly common among older adults in outpatient settings, despite often being preventable [5]. Polypharmacy, the prescription of multiple medications, is a primary factor leading to adverse drug events and untoward drug interactions [6, 7]. The prevalence of polypharmacy among older adults is extremely wide-ranging, depending on age, current health status, health care setting, and geography [7]. Epidemiological data suggest that polypharmacy may affect more than one-third of older adults worldwide [8, 9]. Moreover, there is a relationship between polypharmacy and high sarcopenia prevalence, and together, they present a greater risk of frailty [10–12]. However, although polypharmacy constitutes a predictor of several adverse clinical outcomes among older adults, including mortality, a systematic review failed to demonstrate this association because key covariates were not considered [6]. Furthermore, as clinical evidence from longitudinal studies regarding the association between polypharmacy and muscle wasting is lacking, it remains unclear whether widely prescribed medications associated with muscle wasting affect sarcopenia development [13, 14]. Thus, data on the longitudinal contribution of the number of prescribed medications to sarcopenia development among those who do not require long-term care are insufficient.

Polypharmacy is considered a “necessary evil” because several clinical conditions necessitate the use of multiple drugs [15]. Thus, it is important to consider not only the number of medications but also the use of potentially inappropriate medications (PIMs) according to individual health conditions. The Japan Geriatrics Society published guidelines reflecting the current medical and medication situation in Japan, which were revised in 2015 as the “Screening Tool for Older Person’s Appropriate Prescriptions for the Japanese” (STOPP-J) [16]. Among Japanses older adults, the prevalnce of PIMs determined by STOPP-J was 41.9%, with higher frequencies among older adults, long-term care insurance users, and polypharmacy [17].Notably, some oral drugs such as statins, sulfonylureas (reiterated), and glinides may be associated with muscle wasting [13]. Moreover, age-associated physiological changes can alter drug disposition, and pathophysiological changes in older adults at risk of developing sarcopenia (e.g., body weight, body composition, protein synthesis) may lead to even greater heterogeneity in pharmacokinetics [18]. However, whether polypharmacy or PIM use is negatively associated with new-onset sarcopenia in community-dwelling older adults remains unclear.

Understanding whether the number of prescribed medications is related to sarcopenia development in community-dwelling older adults could facilitate longer and healthier living, even in consideration of the causal effects of PIM use. Thus, in this study, our aim was to evaluate whether polypharmacy, PIM use, or their combination is associated with a higher risk of sarcopenia over a 9-year follow up period in community-dwelling older adults not requiring long-term care.

## Methods

### Study setting and participants

We employed data from a longitudinal prospective cohort study conducted in Kashiwa, Japan, designed to identify important factors affecting healthy aging in community-dwelling older adults [19]. Urban and rural communities are intermingled in this area. In 2012, we randomly selected 12,000 adults aged 65 years and older with no long-term care needs from the registry of Kashiwa, Japan and sent invitations for participation in the study via mail. A total of 2,044 older adults (1013 men and 1031 women) agreed to participate. The participants reflected the age distribution in Kashiwa for each sex. Baseline data were collected between September and November 2012 at welfare and community centers. Exclusion criteria included (i) cognitive impairment (Mini–Mental State Examination [MMSE] score < 18), (ii) sarcopenia at baseline, (iii) an implanted pacemaker (prohibiting bioelectrical impedance analysis for sarcopenia diagnosis), (iv) missing any follow-up studies, and (v) missing data on sarcopenia or medications. Follow-ups in 2013, 2014, 2016, 2018, and 2021 were performed to examine the longitudinal association between prescribed medications and new-onset sarcopenia. The study followed the Strengthening the Reporting of Observational Studies in Epidemiology (STROBE) reporting guidelines for cohort studies.

The ethics committee of the University of Tokyo Life Science Research Center approved the study protocol (#21–192). Written informed consent was obtained from all participants. This study was conducted in accordance with the tenets of the Declaration of Helsinki.

### Prescribed medications and chronic diseases

Currently prescribed medication names and numbers were determined via face-to-face interviews conducted by trained nurses using a standardized questionnaire. Participants who used a prescription record note were requested to bring the note during assessment. In this study, we defined ≥ 5 drug prescriptions as polypharmacy. The number of prescribed medications were reassessed in all follow-up surveys. The number of prescribed medications was re-examined at all follow-up visits and excluded from polypharmacy when the number of prescribed medications major decreased; PIMs were defined as STOPP-J-listed drugs or potentially muscle-wasting drugs. The investigated medications were thus classified as “PIMs” or “not PIMs.”

STOPP-J drugs were classified into 19 categories and 28 subcategories; the categories consisted of antipsychotics, hypnotics, antidepressants, sulpiride, anti-Parkinson drugs, steroids, antithrombotic drugs (antiplatelet drugs and anticoagulants), digitalis, diuretics, β-blockers, α-blockers, first-generation H1 receptor antagonists, H2 receptor antagonists, antiemetics, laxatives, antidiabetic drugs, insulin, overactive bladder medications, and non-steroidal anti-inflammatory drugs (NSAIDs). As antipsychotics, selective serotonin reuptake inhibitor antidepressants, steroids, digitalis, β-blockers, and laxatives are considered PIMs in STOPP-J only in certain cases or in certain patient subsets (e.g., laxative use among patients with impaired renal function); they were excluded from this study [16]. Sodium–glucose cotransporter 2 (SGLT2) inhibitors were also excluded as they were not available in Japan at study initiation. We also evaluated the use of statins, sulfonylureas (reiterated), and glinides as drugs potentially associated with muscle wasting [13].

Data on current chronic diseases (hypertension, diabetes mellitus, dyslipidemia, osteoporosis, malignant neoplasm, stroke, chronic renal failure, and heart disease) were also obtained during the interviews, with comorbidity defined as the presence of two or more of these.

### Sarcopenia

According to the Asian criteria and cut-off threshold (Asian Working Group of Sarcopenia 2019) [16], we diagnosed sarcopenia as low appendicular skeletal muscle mass with concomitant low muscle strength or physical function. Bioimpedance analyses were used to assess low appendicular skeletal muscle mass, defined as < 7.0 and < 5.7 kg/m^2^ for men and women, respectively, using the InBody 420 body composition analyzer (InBody Japan, Tokyo, Japan) [20]. Low muscle strength (< 28 and < 18 kg, respectively) was measured using a Smedley-type grip strength meter (Grip D dynamometer; Takei Scientific Instruments Co., Ltd., Niigata, Japan) [20]. Low physical function, defined as a normal walking speed of < 1.0 m/s, was determined by measuring the time required to travel 5 m between 11 m lanes [20]. Sarcopenia was remeasured in all follow-up surveys.

### Covariates

Covariates included age, sex, body mass index (BMI), education level (college degree or less), living arrangement (alone vs. together), annual income, cognitive function determined using MMSE [21], depressive symptoms determined using the Geriatric Depression Scale-15 (GDS-15) [22], disability of instrumental activities daily living (IADL) [23], exercise habit (at least once weekly during leisure time) evaluated using the Global Physical Activity Questionnaire [24], daily food diversity, and current alcohol habits (responding “yes” to the question “Do you drink alcohol?”). We also collected data on biochemical parameters (i.e., serum albumin, total cholesterol, hemoglobin, C-reactive protein, platelet count, fasting blood glucose, and systolic/ diastolic blood pressure) via medical interviews and blood tests.

### Statistical analysis

All statistical analyses were performed using IBM SPSS version 29.0 (IBM Japan, Tokyo, Japan). A two-sided *P* value of < 0.05 was considered to indicate statistically significant difference. Data are presented as the mean (± standard deviation) or median (interquartile range) for quantitative measures and as the number of subjects (percentage) for all qualitative measures.

Baseline differences in variables among those with/without new-onset sarcopenia were analyzed using the χ^2^ test or Fisher’s exact test for categorical variables, and unpaired *t*-test or Mann–Whitney U test for continuous variables. We assessed the baseline association between the number of prescribed medications and PIM use via multivariate logistic regression.

To explore the association between prescribed medications and sarcopenia onset, we assessed the longitudinal association between prescribed medications and sarcopenia onset using the Cox proportional hazards model. We calculated the hazard ratios (HRs) and 95% confidence interval (CI) using a bivariate model and a multivariable model, wherein the ratios were adjusted according to the variation in the following continuous variables: age (years), BMI (kg/m^2^), cognitive function (MMSE score), depressive symptoms (GDS-15 score), and daily food diversity score. Ratios were also adjusted baseline status for sex, education level (college degree or less), low annual income (either ≥ or < 1.4 million yen per household for men, and 1.2 million yen for women), living arrangement (alone vs. together), exercise habit (yes/no), current alcohol habit (yes/no), and individual chronic conditions (i.e., hypertension, diabetes mellitus, dyslipidemia, osteoporosis, malignant neoplasm, stroke, chronic renal failure, heart disease, and disability of IADL). Multiple imputation using fully conditional specification (chained equations) was applied to impute the missing values for covariates, and 10 datasets were created.

## Results

### Study participants

A total of 1,549 participants (mean age, 72.5 ± 5.5 years; 49.1% women) were eligible for participation in the present study. Of the 2,044 participants that completed the baseline assessment, 216 were excluded because they failed to meet the inclusion criteria (sarcopenia at baseline, n = 168; pacemaker or missing items; n = 48). Over the 9-year follow-up, 279 individuals were absent from all follow-ups. Over the 9-year follow-up (median year and interquartile range, 6.0 [4.0–9.0] years), 230 individuals (14.8%) experienced sarcopenia development, as determined using criteria from the Asian Working Group of Sarcopenia (AWGS) 2019 (Additional File 1).

### Baseline characteristics and outcomes

Table 1 presents the baseline characteristics of the study participants classified according to new-onset sarcopenia experience. Those with higher age, physical weakness (lower handgrip strength and slower gait speed), psychological deterioration (lower cognitive function and depressive symptoms), and lower serum albumin and hemoglobin levels were more likely to exhibit sarcopenia onset; however, differences were minimal for gait speed, cognitive function, depressive symptoms, and blood test results (lower serum albumin, total cholesterol, and hemoglobin level). Regarding chronic diseases, those with hypertension, heart disease, malignant neoplasm, and comorbidity were more likely to develop sarcopenia. Living alone, low income, lack of exercise, drinking, dyslipidemia, and chronic renal failure tended to be associated with new-onset sarcopenia (*P* < 0.150). No significant differences were found in terms of sex.

**Table 1:** Baseline characteristics of the participants

Baseline conditions	Overall	Sarcopenia development		*P* ^*a*^
		No onset	New onset	
Number of individuals	1,549	1,319	230	
***Basic attributes and daily behavior***				
Age, years	72.5 ± 5.5	72.0 ± 5.0	75.4 ± 5.6	< 0.001
Sex, women	764 (49.3%)	655 (49.7%)	109 (47.4%)	0.54
Education, ≥ college degree	617 (39.8%)	527 (40.0%)	90 (39.0%)	0.88
Living arrangement, alone	170 (11.0%)	137 (10.4%)	33 (14.3%)	0.08
Low yearly income	311 (20.1%)	254 (19.3%)	57 (24.7%)	0.05
Exercise habit	1,257 (81.1%)	1,081 (82.0%)	176 (76.2%)	0.06
Food diversity score	4.0 (2.0–5.0)	4.0 (2.0–5.0)	4.0 (2.0–5.0)	0.55
Alcohol habit, daily	769 (49.6%)	665 (50.4%)	104 (45.0%)	0.15
***Psychological status***				
MMSE score	28.3 ± 1.8	28.4 ± 1.7	27.8 ± 2.0	< 0.001
GDS-15 score	2.0 (0.0–4.0)	1.0 (0.0–4.0)	2.0 (1.0–5.0)	< 0.001
***Physical status (men/women)***				
Body mass index, kg/m^2^	23.4 ± 2.7/22.6 ± 3.2	23.7 ± 2.7/22.8 ± 3.2	22.2 ± 2.6/21.2 ± 2.9	< 0.001
Appendicular SMI, kg/m^2^	7.37 ± 0.6/5.92 ± 0.6	7.46 ± 0.6/6.01 ± 0.6	6.84 ± 0.7/5.40 ± 0.4	< 0.001
Handgrip strength, kg	35.6 ± 5.4/23.0 ± 3.5	36.3 ± 5.3/23.4 ± 3.4	31.5 ± 4.1/20.5 ± 2.7	< 0.001
Usual gait speed, m/s	1.49 ± 0.2/1.49 ± 0.2	1.51 ± 0.2/1.50 ± 0.2	1.40 ± 0.2/1.39 ± 0.2	< 0.001
***Biochemical parameters***				
Serum albumin, g/dL	4.43 ± 0.22	4.44 ± 0.22	4.40 ± 0.22	0.007
Total cholesterol, mg/dL	213 ± 33	213 ± 33	208 ± 34	0.05
Hemoglobin, g/dL	14.0 ± 1.3	14.0 ± 1.3	13.7 ± 1.2	< 0.001
C-reactive protein, mg/dL	0.12 ± 0.3	0.11 ± 0.3	0.14 ± 0.4	0.19
Platelet count, per 10^4^ µL	21.8 ± 5.8	21.8 ± 5.9	21.6 ± 5.4	0.66
***Chronic conditions***				
Hypertension	669 (43.2%)	552 (41.8%)	117 (50.6%)	0.011
Dyslipidemia	620 (40.0%)	518 (39.3%)	102 (44.2%)	0.15
Heart disease	261 (16.8%)	206 (15.6%)	55 (23.8%)	0.002
Malignant neoplasm	230 (14.8%)	183 (13.9%)	47 (20.3%)	0.010
Diabetes mellitus	192 (12.4%)	161 (12.2%)	31 (13.4%)	0.59
Osteoporosis	149 (9.6%)	122 (9.2%)	27 (11.7%)	0.24
Stroke	94 (6.1%)	76 (5.8%)	18 (7.8%)	0.22
Chronic renal failure	12 (0.8%)	8 (0.6%)	4 (1.7%)	0.09
Comorbidity, ≥ 2 diseases	664 (42.9%)	539 (40.9%)	125 (54.3%)	< 0.001
Number of medications	2.0 (0.0–4.0)	1.0 (0.0–4.0)	2.0 (1.0–5.0)	< 0.001
Disablity of IADL	19 (1.2%)	11 (0.83%)	8 (3.5%)	< 0.001

Notes: MMSE, Mini-Mental State Examination; GDS-15, Geriatric Depression Scale-15; SMI, skeletal muscle mass index; IADL, instrumental activiteis daily living

Data are presented as the mean (± standard deviation) or median (interquartile range) for quantitative measures and as the number of subjects (percentages) for all qualitative measures

^a^ Baseline differences in variables among those with/without new-onset sarcopenia were analyzed using the χ^2^ test or Fisher’s exact test for categorical variables and unpaired *t*-test or Mann–Whitney U test for continuous variables

### Prescribed medications and potentially inappropriate medications

The median number of prescribed medications was 2.0 (interquartile range, 0–4; range, 0–17), whereas 428 (27.6%) individuals had no prescribed medications. The frequency of PIM use and the longitudinal association with sarcopenia onset is summarized in Additional File 2. According to the STOPP-J criteria, 436 (28.1%) individuals were prescribed PIMs, and 381 (24.6%) were prescribed potentially muscle-wasting drugs. Benzodiazepines and NSAIDs were the most commonly prescribed drugs matching the STOPP-J criteria; statins were the most commonly prescribed potentially muscle-wasting drugs. Only NSAIDs, muscarinic receptor antagonists, and statins were significantly associated with sarcopenia onset.

The association between the number of prescribed medications and use of PIMs, such as drugs listed in STOPP-J and potentially muscle-wasting drugs, at baseline are summarized in Additional File 3. Compared to the proportion of individuals with one prescription, the proportion of individuals prescribed PIMs increased significantly with an increase in the number of prescriptions, exceeding 50% for individuals with ≥ 4 prescriptions. The inclusion rate of potential muscle-weakening drugs was also significantly higher for individuals prescribed ≥ 5 drugs. Therefore, our definition of ≥ 5 drug prescriptions as polypharmacy, consistent with the definition of a previous study [25], representing 367 (23.7%) participants.

### Prescribed medications and sarcopenia onset

Next, we examined whether polypharmacy, PIM use, and potential muscle-wasting drug use were individually associated with new-onset sarcopenia over the 9-year follow-up. The covariate-adjusted hazard ratios and 95% CIs of sarcopenia onset based on prescribed medications are presented in Table 2. After adjusting for covariates, older adults with polypharmacy tended to have an increased frequency of new-onset sarcopenia (25.6% vs. 11.5%), along with a large adjusted hazard ratio compared with those without polypharmacy. The use of STOPP-J-listed drugs and potentially muscle-wasting drugs tended to be associated with sarcopenia development.

**Table 2:** Longitudinal association of the number of prescribed medications and PIM use with new-onset sarcopenia

Exposure	New-onset sarcopenia		Crude model		Multivariate model^b^	
	No. of cases (%)		HR (95% CI)	*P*	HR (95% CI)	*P*
Polypharmacy, the number of prescribed medications						
< 5 drugs	136 / 1,187	(11.5%)	1.00 (reference)		1.00 (reference)	
≥ 5 drugs	94 / 362	(25.6%)	2.70 (2.08–3.52)	< 0.001	2.00 (1.44–2.77)	< 0.001
Drugs listed in STOPP-J						
No use	140 / 1,115	(12.6%)	1.00 (reference)		1.00 (reference)	
Use	90 / 434	(20.6%)	1.80 (1.38–2.34)	< 0.001	1.44 (1.07–1.95)	0.018
Potentially muscle-wasting drugs						
No use	155 / 1,170	(13.3%)	1.00 (reference)		1.00 (reference)	
Use	75 / 379	(19.7%)	1.49 (1.13–1.96)	0.005	1.48 (1.04–2.09)	0.029
Polypharmacy with or without the use of PIMs^a^						
< 5 drugs without PIM	94 / 844	(11.2%)	1.00 (reference)		1.00 (reference)	
< 5 drugs with PIM	42 / 343	(12.2%)	1.08 (0.75–1.55)	0.694	1.15 (0.77–1.71)	0.487
≥ 5 drugs without PIM	16 / 84	(18.6%)	1.99 (1.17–3.37)	0.011	1.56 (0.81–2.89)	0.299
≥ 5 drugs with PIM	78 / 278	(27.8%)	3.00 (2.22–4.05)	< 0.001	2.35 (1.58–3.51)	< 0.001

*Notes*: HR, hazard ratio; CI, confidence interval; PIM, potentially inappropriate medication; STOPP-J, screening tool for older person’s appropriate prescriptions for the Japanese. Hazard ratios and 95% CI were calculated using the Cox proportional hazards model

^a^ PIMs were drugs listed in STOPP-J or potentially muscle-wasting drugs

^b^ The multivariate model included the following potentially confounding baseline factors: age, sex, education level (college degree or less), low annual income, body mass index, living alone, cognitive function, depressive symptoms, exercise habits, daily food diversity, alcohol habits, and chronic diseases (hypertension, diabetes mellitus, dyslipidemia, osteoporosis, malignant neoplasm, stroke, chronic renal failure, heart disease and disablity of insturmental activities daily living)

Furthermore, we tested whether polypharmacy combined with the use of PIMs (drugs listed in STOPP-J or potential muscle-wasting drugs) was associated with sarcopenia development. Overall, 76.6% of older adults on polypharmacy also utilized PIMs, which was strongly correlated with new-onset sarcopenia. Neither polypharmacy nor PIM use alone was significantly associated with sarcopenia development.

## Discussion

In this longitudinal cohort study, we examined the prescribed medications, chronic diseases, and physical and psychological status of individuals to identify whether polypharmacy is associated with an increased risk of sarcopenia development over a 9-year period among community-dwelling older adults with no long-term care needs. After adjusting for multifaceted confounders, polypharmacy combined with the use of STOPP-J-listed PIMs or potentially muscle-wasting drugs, but not polypharmacy alone, was significantly associated with an increased HR of sarcopenia onset.

To the best of our knowledge, our study provides the first evidence of the possibility of a longitudinal association between polypharmacy combined with PIMs and sarcopenia development among community-dwelling older adults. Similarly, a previous cross-sectional study reported that polypharmacy (≥ 5 medications) in older adults was associated with a 2.2-fold higher prevalence of sarcopenia based on low appendicular lean mass [10]. Our results are consistent with the findings from previous observational studies in other populations showing associations between polypharmacy and adverse geriatric outcomes, such as frailty, sarcopenia, and mortality [6, 10–12, 26].

Several possible explanations exist for the longitudinal association between polypharmacy and new-onset sarcopenia. First, older adults have an increased risk of adverse drug reactions (ADRs) because of age-related changes in pharmacokinetics, such as absorption, bioavailability, distribution, metabolism, and excretion [27]. Previous review informs the impact of chronological aging and various geriatric syndromes on drug disposition, and there is some evidence that changes in body weight and composition as well as protein synthesis affect drug distribution and metabolism [28]. Thus, poor pharmacokinetics may enhance the adverse outcomes of polypharmacy and long-term dosing.

Second, polypharmacy is strongly associated with PIM use [29], prescribing cascades [30], and low adherence [31]. A prescribing cascade occurs when nonspecific signs and symptoms of ADRs are misinterpreted as disease, and further medications are prescribed to treat the misinterpreted conditions. Unfortunately, because the symptoms caused by polypharmacy usually mimic those of aging, such as depressive symptoms [32], cognitive decline [33], and frailty [11, 12], the probability of prescribing cascades is higher among older adults exhibiting frailty. Thus, prescribing cascades can lead to the prescription of PIMs [30]. Our results also showed that individuals with polypharmacy were also prescribed PIMs or potentially muscle-wasting drugs, suggesting that polypharmacy may be linked to overmedication. Participants with new-onset sarcopenia were not only older at baseline but also exhibited lower physical and cognitive function, as well as more depressive symptoms. It is conceivable that psychological characteristics contributed to the lower adherence. These negative influences can explain why polypharmacy is associated with preventable and unplanned hospitalization [6, 34] and ADRs [5, 6, 35]. Our results showed that the risks of sarcopenia did not increase until ≥ 5 medications were used, supporting previous findings that older Japanese adults taking ≥ 6 medications were more likely to experience ADRs [36]. Therefore, in individuals with polypharmacy, the high risk of ADRs associated with age-related changes in pharmacokinetics, PIMs, and low adherence to prescribed medications could explain the increased risks of adverse outcomes.

Furthermore, we found that PIMs or potentially muscle-wasting drug use tended to be associated with increased sarcopenia risk. In addition to medication number and possible inter-drug interactions, the types of drugs used may directly or indirectly affect sarcopenia. For example, some drugs may negatively affect muscles, inducing eating disorders, or may lead to weight loss, which influences body composition [13, 14]. Potentially muscle-wasting drugs, such as statins, can cause muscle toxicity, whereas other PIMs (e.g., glucocorticoids, β blockers, and NSAIDs) may have detrimental metabolic effects. Statins may also induce serious adverse effects, including elevated liver enzymes and skeletal muscle abnormalities, ranging from benign myalgias to severe rhabdomyolysis [13, 14], potentially derived from mitochondrial dysfunction, alterations in apoptosis-related gene expression, protein degradation, and genetic predisposition. Such mechanisms are also involved in sarcopenia pathogenesis and may underlie the association between statins and sarcopenia. Furthermore, the use of PIMs such as benzodiazepine constitutes a risk factor for adverse health outcomes and may be contraindicated for long-term use [37–39]. Therefore, despite the benefits of appropriate medications for older individuals, the prescription of too many medications, including PIMs, may negatively influence geriatric endpoints such as sarcopenia. Consistently, after adjustment for potential confounders, including time-varying factors, we observed that polypharmacy with PIMs was significantly associated with sarcopenia development, thereby explaining the longitudinal association between polypharmacy combined with PIMs and increased sarcopenia risk over a 9-year period.

This study has some limitations. First, although we adjusted for several confounding factors, some biases may have remained owing to unobserved medical conditions or altered renal or liver function. Second, information on the duration of drug treatment or medication adherence was lacking. As such, the exact doses of the prescribed medications were unknown. Third, ADR occurrence, medication underuse, and medication duplication were not assessed. Fourth, the number of medications was evaluated at each follow-up and confirmed that the number of medications did not decrease dramatically, but PIMs could not be evaluated at each follow-up. Fifth, further prospective randomized control trials are necessary to evaluate the potential benefits of de-prescribing on sarcopenia risk. Notably, this study included a sample of community-dwelling older adults who participated through randomization, for whom health awareness is expected to be high, and the population representativeness is relatively high; consequently, the generalizability of the results is high. However, further research is required to determine whether similar results would be obtained in countries with different healthcare supply systems.

## Conclusions

In this longitudinal study, we evaluated whether the number and type of prescribed medications are associated with increased sarcopenia risk among community-dwelling older adults. After adjustment for time-varying confounders, polypharmacy combined with PIM use was associated with an increased risk of sarcopenia onset over a 9-year follow-up period. Our results suggest that the appropriateness of multiple drug prescriptions should be thoroughly evaluated, particularly among individuals with polypharmacy. Efforts to structurally manage and advocate for appropriate drug prescribing, taking into account the characteristics of geriatric syndromes such as sarcopenia, may help prevent sarcopenia.

## Electronic supplementary material

Below is the link to the electronic supplementary material.

Supplementary Material 1

Supplementary Material 2

Supplementary Material 3

## Data Availability

The datasets generated and analyzed during the current study are not publicly available due them containing information that could compromise research participant privacy or consent but are available from the corresponding author on reasonable request.

## List of abbreviations

ADRs: adverse drug reactions
AWGS: Asian Working Group of Sarcopenia
BMI: body mass index
HR: hazard ratio
OR: odds ratio
CI: confidence interval
GDS-15: Geriatric Depression Scale-15
MMSE: Mini–Mental State Examination
NSAID: non-steroidal anti-inflammatory drug
HR: hazard ratio
PIM: potentially inappropriate medication
SGLT2: sodium–glucose cotransporter 2
STOPP-J: Screening Tool for Older Person’s Appropriate Prescriptions for the Japanese
STROBE: Strengthening the Reporting of Observational Studies in Epidemiology.
