# Supplementary material for: Polypharmacy with potentially inappropriate medications as a risk factor of new onset sarcopenia among community-dwelling Japanese older adults: a 9-year Kashiwa cohort study
Source: BMC Geriatr. 2023 Jun 26;23:390. doi: 10.1186/s12877-023-04012-y (PMC10294366; doi:10.1186/s12877-023-04012-y)
Supplement: Supplementary file 1 — Supplementary Material 1 [file 12877_2023_4012_MOESM1_ESM.docx]

| **Additional File 1.** Number of cases from each survey | | | |
| --- | --- | --- | --- |
|  | Total number of measurements | Sarcopenia development  Number of cases (%) | |
| Baseline survey in 2012 | 1,549 | 0 | (0.0%) |
| First follow-up survey in 2013 | 1,402 | 56 | (4.0%) |
| Second follow-up survey in 2014 | 1,200 | 45 | (3.8%) |
| Third follow-up survey in 2016 | 882 | 45 | (5.1%) |
| Fourth follow-up survey in 2018 | 808 | 59 | (7.3%) |
| Fifth follow-up survey in 2021 | 415 | 55 | (13.3%) |
| Cumulative number of individuals with new onset sarcopenia^a^ | 1,549 | 230 | (14.8%) |
| Notes: The median value and interquartile range of follow-up years, 6.0 (4.0–9.0) years.  Sarcopenia was determined based on the Asian Working Group of Sarcopenia 2019 criteria.  ^a^, Thirty individuals were excluded from the cumulative number due to multiple onset. | | | |
