# Supplementary material for: Polypharmacy with potentially inappropriate medications as a risk factor of new onset sarcopenia among community-dwelling Japanese older adults: a 9-year Kashiwa cohort study
Source: BMC Geriatr. 2023 Jun 26;23:390. doi: 10.1186/s12877-023-04012-y (PMC10294366; doi:10.1186/s12877-023-04012-y)
Supplement: Supplementary file 2 — Supplementary Material 2 [file 12877_2023_4012_MOESM2_ESM.docx]

| **Additional File 2.** Frequency of PIM use at baseline and longitudinal association with sarcopenia development | | | | | |
| --- | --- | --- | --- | --- | --- |
| Category | Subcategory | Overall  (n = 1,549) | Sarcopenia development | | *P^a^* |
|  |  |  | No onset  (n = 1,319) | New onset (n = 230) |  |
| **Drugs listed in STOPP-J** | | 436 (28.1%) | 346 (26.2%) | 90 (39.0%) | <0.001 |
| Hypnotics | Benzodiazepines | 138 (8.9%) | 114 (8.6%) | 24 (10.0%) | 0.38 |
|  | Non-benzodiazepine hypnotics | 51 (3.3%) | 39 (3.0%) | 12 (5.2%) | 0.11 |
| Antidepressants | Tricyclic antidepressants | 7 (0.5%) | 4 (0.3%) | 3 (1.3%) | 0.07 |
| Sulpiride | Sulpiride | 2 (0.1%) | 1 (0.1%) | 1 (0.4%) | 0.28 |
| Anti-Parkinson drugs | Anticholinergic drugs | 4 (0.3%) | 3 (0.2%) | 1 (0.4%) | 0.47 |
| Antithrombotic drugs | Antiplatelet drugs including aspirin^b^ | 247 (15.9%) | 188 (14.3%) | 59 (25.5%) | 0.08 |
|  | Multiple antithrombotic drugs | 38 (2.5%) | 31 (2.4%) | 7 (3.0%) | 0.49 |
| Digitalis | Digoxin | 5 (0.3%) | 3 (0.2%) | 2 (0.9%) | 0.16 |
| Diuretics | Loop diuretics | 21 (1.4%) | 17 (1.3%) | 4 (1.7%) | 0.54 |
|  | Aldosterone antagonists | 7 (0.5%) | 7 (0.5%) | 0 (0.0%) | 0.60 |
| α-Blockers | Nonselective | 10 (0.6%) | 8 (0.6%) | 2 (0.9%) | 0.65 |
| H_1_ receptor antagonists | First-generation | 9 (0.6%) | 7 (0.5%) | 2 (0.9%) | 0.63 |
| H_2_ receptor antagonists | H_2_ receptor antagonists | 54 (3.5%) | 41 (3.1%) | 13 (5.6) | 0.08 |
| Antiemetics | Antiemetic drugs (0) | 0 (0.0%) | 0 (0.0%) | 0 (0.0%) | N/A |
| Antidiabetic drugs | Sulfonylureas | 62 (4.0%) | 51 (3.9%) | 11 (4.8%) | 0.51 |
| Antidiabetic drugs  Insulin | Biguanides | 41 (2.6%) | 32 (2.4%) | 9 (3.9%) | 0.19 |
|  | Thiazolidine derivatives | 39 (2.5%) | 31 (2.4%) | 8 (3.5%) | 0.36 |
|  | α-Glucosidase inhibitors | 35 (2.3%) | 26 (2.0%) | 9 (3.9%) | 0.09 |
| Overactive bladder medications | Oxybutynin (oral) | 0 (0.0%) | 0 (0.0%) | 0 (0.0%) | N/A |
|  | Muscarinic receptor antagonists | 19 (1.2%) | 11 (0.8%) | 8 (3.5%) | 0.001 |
| NSAIDs | NSAIDs | 98 (6.3%) | 71 (5.4%) | 27 (11.7%) | 0.011 |
| **Potentially muscle-wasting drugs** | | 381 (24.6%) | 156 (11.8%) | 75 (19.7%) | 0.005 |
| Statins | | 331 (21.4%) | 264 (20.0%) | 67 (29.0%) | 0.002 |
| Sulfonylureas (reiterated) | | 62 (4.0%) | 51 (3.9%) | 11 (4.8%) | 0.51 |
| Glinides | | 21 (1.4%) | 16 (1.2%) | 5 (2.2%) | 0.22 |
| Notes: PIM, potentially inappropriate medication; NSAID, non-steroidal anti-inflammatory drug; N/A, not available.  ^a^ Baseline differences in variables among those with/without new-onset sarcopenia were analyzed using the χ^2^ test or Fisher’s exact test.  ^b^ STOPP-J criteria do not apply to stand-alone use. | | | | | |
