# Supplementary material for: Polypharmacy with potentially inappropriate medications as a risk factor of new onset sarcopenia among community-dwelling Japanese older adults: a 9-year Kashiwa cohort study
Source: BMC Geriatr. 2023 Jun 26;23:390. doi: 10.1186/s12877-023-04012-y (PMC10294366; doi:10.1186/s12877-023-04012-y)
Supplement: Supplementary file 3 — Supplementary Material 3 [file 12877_2023_4012_MOESM3_ESM.docx]

| **Additional File 3.** Association of the number of prescribed medications with PIM use | | | | | | | |
| --- | --- | --- | --- | --- | --- | --- | --- |
| Number of medications | Overall  N (%) | PIM use at baseline | | | | | |
|  |  | Drugs listed in STOPP-J | | | Potentially muscle-wasting drugs | | |
|  |  | n (%) | aOR (95% CI) ^a^ | *P* | n (%) | aOR (95% CI) ^a^ | *P* |
| Total | 1,549 (100%) | 436 (28.1%) |  |  | 381 (24.6%) |  |  |
| 0 | 428 (27.6%) |  |  |  |  |  |  |
| 1 | 210 (13.6%) | 26 (12.4%) | 1.00 (reference) |  | 47 (22.4%) | 1.00 (reference) |  |
| 2 | 221 (14.3%) | 40 (18.1%) | 1.78 (1.02–3.12) | 0.052 | 52 (23.5%) | 1.17 (0.68–2.02) | 0.36 |
| 3 | 176 (11.4%) | 49 (27.8%) | 3.16 (1.80–5.56) | <0.001 | 59 (33.5%) | 1.60 (0.91–2.81) | 0.11 |
| 4 | 155 (10.0%) | 83 (53.5%) | 8.72 (4.87–15.38) | <0.001 | 53 (34.2%) | 1.53 (0.85–2.75) | 0.13 |
| 5 | 100 (6.5%) | 52 (52.0%) | 9.15 (4.95–17.20) | <0.001 | 50 (50.0%) | 2.89 (1.51–5.55) | 0.003 |
| 6 | 95 (6.1%) | 52(54.7%) | 9.50 (4.95–18.25) | <0.001 | 39 (41.1%) | 2.09 (1.05–4.16) | 0.040 |
| 7 | 57 (3.7%) | 42 (73.7%) | 25.72 (11.66–56.73) | <0.001 | 24 (42.1%) | 2.19 (1.01–4.95) | 0.040 |
| ≥ 8 | 115 (7.4%) | 92 (80.0%) | 28.64 (14.16–57.91) | <0.001 | 57 (49.6%) | 2.72 (1.34–5.52) | 0.017 |
| Notes: PIM, potentially inappropriate medication (drugs listed in STOPP-J and potentially muscle-wasting drugs); aOR, adjusted odds ratio; 95% CI, 95% confidence interval.  ^a^; OR and 95% CI were calculated using logistic regression for PIM use with the number of prescribed medications. Ratio and 95% CI were adjusted for the following potential confounders: age, sex, education level (college degree or less), low annual income (either ≥ or <1.4 million yen per household for men and 1.2 million yen per household for women), body mass index, living alone, cognitive function, depressive symptoms, exercise habits, daily food diversity, alcohol habits, and chronic diseases (hypertension, diabetes mellitus, dyslipidemia, osteoporosis, malignant neoplasm, stroke, chronic renal failure, and heart disease). | | | | | | | |
